# Anti-Inflammatory Mechanisms of Fucoidans to Treat Inflammatory Diseases: A Review

**DOI:** 10.3390/md19120678

**Published:** 2021-11-28

**Authors:** Kalu K. Asanka Sanjeewa, Kalahe H. I. N. M. Herath, Hye-Won Yang, Cheol Soo Choi, You-Jin Jeon

**Affiliations:** 1 Department of Biosystems Technology, Faculty of Technology, University of Sri Jayewardenepura, Pittpana, Homagoma 10200, Sri Lanka; asanka.sanjeewa@sjp.ac.lk; 2 Department of Biosystems Engineering, Faculty of Agriculture and Plantation Management, Wayamba University of Sri Lanka, Makandura, Gonawila 60170, Sri Lanka; madushaniherath@wyb.ac.lk; 3 Department of Marine Life Science, Jeju National University, Jeju 63243, Korea; koty221@jejunu.ac.kr; 4 Korea Mouse Metabolic Phenotyping Center, Lee Gil Ya Cancer and Diabetes Institute, Gachon University, Incheon 21999, Korea; 5 Marine Science Institute, Jeju National University, Jeju 63333, Korea

**Keywords:** fucoidans, brown seaweeds, inflammation, MAPKs, chemokines, cytokines

## Abstract

Fucoidans are sulfated heteropolysaccharides found in the cell walls of brown seaweeds (Phaeophyceae) and in some marine invertebrates. Generally, fucoidans are composed of significant amounts of L-fucose and sulfate groups, and lesser amounts of arabinose, galactose, glucose, glucuronic acid, mannose, rhamnose, and xylose. In recent years, fucoidans isolated from brown seaweeds have gained considerable attention owing to their promising bioactive properties such as antioxidant, immunomodulatory, anti-inflammatory, antiobesity, antidiabetic, and anticancer properties. Inflammation is a complex immune response that protects the organs from infection and tissue injury. While controlled inflammatory responses are beneficial to the host, leading to the removal of immunostimulants from the host tissues and restoration of structural and physiological functions in the host tissues, chronic inflammatory responses are often associated with the pathogenesis of tumor development, arthritis, cardiovascular diseases, diabetes, obesity, and neurodegenerative diseases. In this review, the authors mainly discuss the studies since 2016 that have reported anti-inflammatory properties of fucoidans isolated from various brown seaweeds, and their potential as a novel functional material for the treatment of inflammatory diseases.

## 1. Introduction

**Figure 1:**
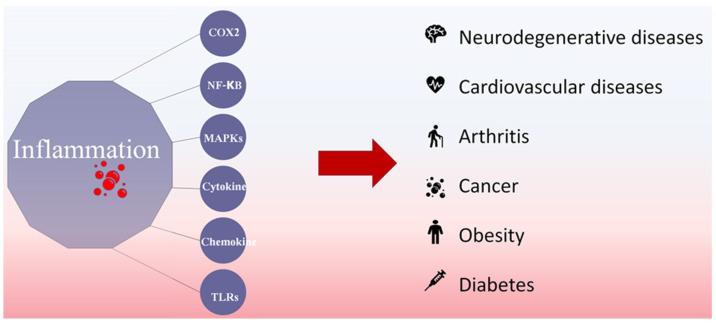
Disease conditions linked with chronic inflammatory responses.

Inflammation is a complex immune response that protects the organs from infection and tissue injury. Controlled inflammatory responses are beneficial to the host, resulting in the removal of immunostimulants from the host tissues and restoration of structural and physiological functions [1]. However, inflammatory responses are also associated with the pathogenesis of tumor development, arthritis, cardiovascular diseases, diabetes, obesity, and neurodegenerative diseases due to the upregulated expression of cytokines, chemokines, nuclear transcription factor-kappa B (NF-κB), mitogen-activated protein kinase (MAPK), and Toll-like receptors (Figure 1) [2,3]. The characteristic features of inflammation include heat, swelling, redness, pain, and loss of tissue functions. Studies have shown that up to 20% of human cancers are related to chronic, unresolved inflammation caused by viral and bacterial infections, exposure to irritants, and autoimmune diseases [4]. Thus, inhibition of excessive inflammatory responses is a critical factor so as to maintain a well-balanced immune system and human health [3]. 

Fucoidans are sulfated heteropolysaccharides found in brown seaweeds (Phaeophyceae), localized in the cell wall and marine invertebrates. In particular, fucoidans isolated from brown seaweeds have gained considerable attention in recent decades owing to their promising bioactive properties [5]. Specifically, the antioxidant, immunomodulatory, anti-inflammatory, and anticancer properties of fucoidans make them ideal ingredients for use in functional products [5,6,7]. Fucoidans are extracted from different brown seaweeds such as *Ecklonia cava*, *Laminaria japonica*, *Sargassum* spp, *Fucus vesiculosus*, *Fucus evanescens*, and *Undaria pinnatifida* [8,9,10]. In addition to brown seaweeds, Echinoderms, (starfish, sea urchins, and sea cucumbers) are also found to produce fucoidan [11]. Sea cucumbers species that have been explored for fucoidan production include *Apostichopus japonicus, Holothuria tubulosa*, and *Stichopus japonicus* [12,13]. However, reproductive rates of seaweeds are higher than reproductive rates of sea cucumbers, which can be considered moderate, compared with other invertebrates. Therefore, in terms of sustainability, commercial fucoidan producers should focus more on extraction from seaweeds than Echinoderms.

The structures and bioactivities of fucoidans are dependent on the species, harvesting season, harvest location, maturity of the plant, and extraction method [8,14]. The basic structure of fucoidans consists of a sulfated fucose backbone and includes small quantities of monosugars, including galactose, mannose, glucose, arabinose, uronic acids, and xylose [15]. The molecular weight of fucoidans ranges between 40 and 1600 kDa [14]. However, due to the differences in structural and specific properties of fucoidans, most of the studies reporting the bioactive properties of fucoidans have failed to characterize the structural properties of the isolated fucoidans. Therefore, difficulties have been encountered in the formulation of fucoidans for specific functional activities and in drawing conclusions regarding the identified functional properties [15]. This review outlines the recent findings that are related to the anti-inflammatory properties of fucoidans, isolated from different brown seaweeds, and their potential as novel molecules for the treatment of inflammatory diseases. 

## 2. Anti-Inflammatory Mechanisms of Fucoidans

### 2.1. COX2 Inhibition of Fucoidan

**Figure 2:**
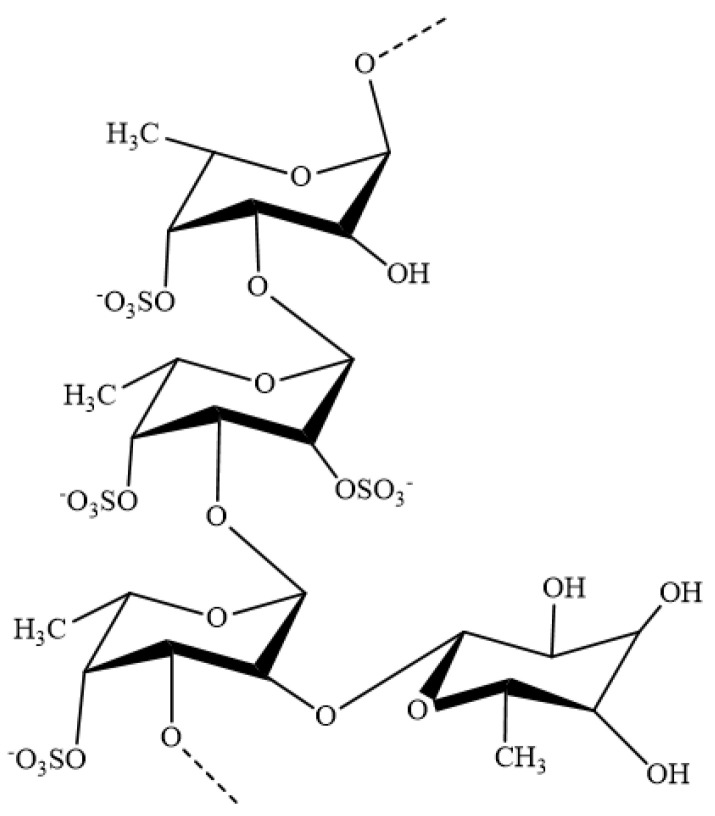
General structure of fucoidans isolated from brown seaweeds.

Cyclooxygenase-2 (COX2) is a well-known inflammatory mediator secreted by a number of cells [16]. The secretion of COX2 in macrophages is triggered by inflammatory stimuli, and the action of COX2 leads to the conversion of arachidonic acid to prostaglandins (PGs). The most abundant PG in the human body is prostaglandin E2 (PGE2) [17]. According to recent findings, PGE2 is the principal mediator of inflammatory diseases, such as rheumatoid arthritis and osteoarthritis. In most cases, nonsteroidal anti-inflammatory medications and selective COX2 inhibitors are used to treat arthritis and osteoarthritis, as they are capable of inhibiting upregulated PGE2 secretion [18]. Thus, COX2 inhibitors have a specific value in the functional food industry. In this section, we discuss several studies reporting COX2 inhibitory properties of fucoidans (Figure 2). 

Studies have shown that the dysfunction of chondrocytes, such as degradation of type II collagen and inflammatory responses, is linked to joint-related diseases, such as osteoarthritis and rheumatoid arthritis, as well as physicomechanical functioning of tissues [19]. Phull and Kim (2017) evaluated the protective effects of fucoidans isolated from *U. pinnatifida*, using rabbit articular chondrocytes. According to the authors, fucoidans (2.5–100 µg/mL) downregulated the levels of COX2 in chondrocytes, in a dose-dependent and time-dependent manner [20]. In addition, Phull et al. (2017) reported the possibility of fucoidans, isolated from *U. pinnatifida*, to ameliorate clinical symptoms in arthritic rats. The authors found that downregulation of COX2 by fucoidans may be correlated with antiarthritic effects [20]. Similarly, Garcia et al. (2019) reported that fucoidans (5 µg/mL), isolated from *F. vesiculosus*, *U. pinnatifida*, and *Macrocystis pyrifera,* have the potential to inhibit interleukin (IL)-1β-induced-COX2 secretion from chondrocytes and synoviocytes [21]. 

Jeong et al. (2016) reported the ability of fucoidans to inhibit lipopolysaccharide (LPS)-induced PGE2 secretion in RAW 264.7 macrophages, as well as COX2 secretion in zebrafish embryos. According to the authors, fucoidan treatment at doses up to 100 µg/mL inhibited LPS-induced inflammatory responses, without being toxic to RAW 264.7 macrophages and zebrafish embryos [22]. Fucoidans also inhibited LPS-induced PGE2 production in macrophages by downregulating COX2 secretion. Park et al. (2017) investigated the anti-inflammatory properties of fucoidans in vivo and in vitro, using an LPS solution prepared from *Porphyromonas gingivalis*. According to the authors, fucoidan treatment led to reduced levels of COX2 levels in six-week-old female BALB/c mice and RAW 264.7 macrophages treated with LPS-alone [23]. The COX2 inhibitory properties of commercial-grade fucoidans against gamma-irradiated male Wistar albino rats were assessed by Azab et al. (2017). The authors reported that rats administered fucoidans had improved anti-inflammatory properties, compared with positive controls [24]. COX2 inhibitory properties of fucoidans isolated from *Turbinaria ornate* against LPS-activated RAW 264.7 macrophages and zebrafish have also been reported [25]. COX2 inhibitory effects of fucoidans isolated from *Chnoospora minima* and *Sargassum polycystum* have also been reported by Fernando et al. (2018) using LPS-activated RAW 264.7 macrophages [26,27]. In addition, Luo et al. (2019) reported the COX2 inhibitory effect of fucose-containing polysaccharides (373 kDa), isolated from *S. thunbergii* (STSP-1). The authors found that treatment with STSP-1 downregulated LPS-activated *COX2* expression in RAW 264.7 macrophages at concentrations of 75 and 150 μg/mL [28]. In a recent study by Wang et al. (2020), the COX2 inhibitory effect of fucoidans, isolated from *H. fusiforme*, was demonstrated. The authors observed a significant reduction in COX2 protein expression in LPS-activated RAW 264.7 macrophages after treatment with fucoidans [29]. Additionally, Pozharitskaya et al. (2020) also reported the COX2 inhibitory effect of fucoidan (735 kDa) isolated from *Fucus vesiculosis* [30]. According to the results, fucoidan inhibits the COX2 with an IC_50_ value of 4.3 μg/mL. Taken together, these results suggest that fucoidans have the potential to improve inflammatory responses via blocking the conversion of arachidonic acid into PGE2 through the inhibition of COX2 (refer to Table 1). However, inhibition of COX2 can be a result of inhibition of one or several inflammatory intra- and intercellular signal cascades. Therefore, in subsequent sections of the review, the anti-inflammatory mechanisms of fucoidans are discussed.

### 2.2. NF-κB Inhibition of Fucoidan

NF-κB is a transcription factor found in nucleated cell types, which regulates gene expression induced by diverse stress signals, such as signal transducer and activator of transcription (STAT), MAPKs nuclear hormone receptors, cytokines (IL-1β, tumor-necrosis factor-α [TNF-α], and IL-6), inducible nitric oxide synthase (iNOS), and COX2 [31,32]. NF-κB is reported in multiple forms, with the most prevalent being inactive dimer combination of its p50 and p65 subunits. Under unstressed conditions, NF-κB exists in an inactive form, composed of a dimeric form of the transcription factor, and binds with the inhibitor of κB (I-κB), which maintains NF-κB in its inactive form [33]. Phosphorylation of I-κB results in the release of cytoplasmic p50 and p65 from its inactive form and allows them to translocate into the nucleus [34]. Thus, inhibition of NF-κB signal transduction is one of the possible mechanisms underlying the downregulation of chronic inflammatory responses. Therefore, in this section, we discuss several studies that have reported the inhibitory effects of fucoidans on NF-κB (Table 2).

In a recent study, Bai et al. (2020) determined the anti-inflammatory properties of fucoidan isolated from *Fucus vesiculosus* against *Schistosoma japonicum* induced inflammation in the *S. japonicum*-infected mice model. According to the authors, fucoidan decreased phospho-p65 protein and the mRNA levels of pro-inflammatory cytokines (IL-6, IL-12, and TNF-α) in the livers from fucoidan-treated *S. japonicum*-infected mice. According to the authors, natural fucoidan might have the potential to develop novel therapeutics against hepatic disease caused by parasitic chronic infection [35].

Hu et al. (2020) observed the NF-κB inhibitory effect of fucoidans, isolated from *Kjellmaniella crassifolia,* cultured in Dalian, northern China, on aspirin-induced gastric ulcers in a Wistar rat model [36]. The results, analyzed using RT-PCR and Western blotting, indicated that fucoidans suppressed aspirin-induced NF-κB activation via stabilization of IκB-α in the Wistar rat model, thereby inducing the downregulation of *COX2* and *iNOS* expression. In a study by Hu et al. (2019), the NF-κB inhibitory properties of fucoidans were demonstrated in a myocardial infarction rat model. Results from their study showed that fucoidans downregulated IκB degradation and subsequent phosphorylation of NF-κB p65 in rat myocardial tissues [37]. Zheng et al. (2018) determined the protective effect of low molecular weight (LMW) fucoidans in obese diabetic db/db mice against nonalcoholic fatty liver disease and observed a marked increase in NF-κB expression in the nuclear proteins of db/db hepatic tissues. In addition, LMW fucoidan treatment significantly attenuated the upregulated NF-κB phosphorylation, compared with that in the vehicle-treated group [38]. Hai-Lan et al. (2019) also confirmed the NF-κB-p65 inhibitory effect of fucoidans, isolated from *S. weizhouense*. According to the authors, porcine circovirus type 2-induced *NF-κB-p65* gene transcription in Kunming inbred mice [39]. Similarly, Zhang et al. (2018) demonstrated the NF-κB-p65 inhibitory effects of fucoidans isolated from *L*. *japonica* in adenine-induced hyperuricemia mice. The authors found that the expression and activation of NF-κB-p65 were promoted in the kidneys of adenine-treated mice but downregulated after fucoidans administration [40]. In a recent study by Xue et al. (2019), the NF-κB-p65 inhibitory effects of fucoidans were observed in 7-week-old NOD diabetic mice. The authors found weaker NF-κB-p65 expression in the pancreatic tissues of fucoidan-treated groups [41]. Nguyen et al. (2016) reported the anti-inflammatory effects of sodium alginate/gelatin porous scaffolds merged with fucoidans in murine microglial BV2 cells and found that fucoidans have the potential to inhibit the activation and translocation of NF-κB-p65 protein levels in activated BV2 cells and subsequent inhibition of NO, ROS, and PGE2 production [42]. In a recent study, we found that fucoidans isolated from *Padina strumsonii* have the potential to protect macrophages against LPS-activated NF-κB activation and translocation. Specifically, we found that fucoidans isolated from *P. commersonii* were capable of inhibiting phosphorylation of IKK-α, IKβ-α, and subsequent phosphorylation of NF-κB-p65 and p50 phosphorylation in the cytosol [43]. In addition, Sanjeewa et al. (2018) reported the NF-κB inhibitory properties of fucoidans isolated from *S. horneri*. According to the authors, fucoidans alleviated LPS-activated IKβ-α phosphorylation and subsequent upregulation of iNOS and COX2 in LPS-exposed RAW 264.7 macrophages [44]. 

### 2.3. MAPK Inhibition of Fucoidan

The MAPK family of signaling molecules plays an important role in many cellular functions, including control of proliferation and stress responses. Similar to NF-κB, MAPKs integrate signals from various stimuli through the regulated and sequential transfer of covalent modifications (phosphorylation) of signaling intermediates. MAPKs consist of three proteins—namely, p38 kinases, c-Jun N-terminal kinases (JNK), and extracellular regulated protein kinase 1/2 (ERK1/2) [45]. It has been reported that ERK, JNK, and p38 can be activated by several intracellular and extracellular factors, such as pro-inflammatory cytokines, oxidative stress, genotoxicity, osmotic, and hypoxia. Activated MAPKs are found to upregulate inflammatory responses such as elevated pro-inflammatory cytokine secretion and NF-κB activation in mammalian cells [46].

Several studies have reported that fucoidans isolated from brown seaweeds have the potential to inhibit MAPK phosphorylation (Table 2). Here, we discuss the MAPK inhibitory properties of fucoidans isolated from brown seaweeds. Che et al. (2017) evaluated the MAPK inhibitory effect of commercial-grade fucoidans, purchased from Sigma-Aldrich Corporation (St. Louis, MO, USA), in a cerebral ischemia–reperfusion injured rat model [47]. According to the authors, fucoidan treatment inhibited the phosphorylation of MAPKs (ERK, JNK, and p38) in ischemia–reperfusion injured rats. In addition, the inhibitory properties of commercial-grade fucoidans (*U. pinnatifida*), purchased from Sigma-Aldrich, were reported by Choo et al. (2016). According to the authors, inhibition of MAPKs (ERK and p38) affected the proliferation of DU-145 cancer cells [48]. In a recent study, we also stated that fucoidans isolated from *S. horneri* have the potential to inhibit LPS-activated inflammation in RAW 264.7 macrophages by inhibiting phosphorylation of ERK and JNK [49]. In our study, we observed that the bioactivity of fucoidans was dependent on several factors, including fucose and sulfate residues in the fucoidans structure. Wu et al. (2016) explored the underlying mechanisms related to the NO inhibitory effect of fucoidans isolated from *S. cristaefolium*. The authors found that fucoidan treatment (10–400 µg/mL) could alleviate MAPK (ERK, JNK, and p38) phosphorylation and subsequent iNOS expression in LPS-activated RAW 264.7 macrophages [50]. Nie et al. (2017) reported the protective effects of fucoidans against hyperoxic lung injury in a Balb/c mouse model. The authors assessed hyperoxia-induced phosphorylation of ERK1/2 in mouse lung tissues, with or without atomization inhalation of fucoidans (100 µg/mL), for 36 h. Results from the Western blot analysis showed that hyperoxia significantly increased phosphorylation of ERK1/2 in the lung tissues. However, phosphorylation of ERK1/2 in the lung tissues was significantly reduced following treatment with fucoidans [51].

### 2.4. Cytokine Secretion Modulators

Cytokines are small proteins secreted by the cells and have specific roles in infection, immune responses, inflammation, and trauma. There are two groups of cytokines—namely, anti-inflammatory and pro-inflammatory cytokines. Pro-inflammatory cytokines (IL-1β, IL-6, and TNF-α) act to worsen the existing disease, whereas anti-inflammatory cytokines (IL-4, IL-10, IL-11, and IL-13) reduce inflammation and promote healing [54,55]. Recently, AlKahtane et al. (2019) reported the pro-inflammatory cytokine inhibitory effects of fucoidans, isolated from *L. japonica* (500 mg/capsule, purchased from Absunutrix Lyfetrition (Pennsylvania, PA, USA)), in Swiss albino mice injected with microcystin-LR (10 μg/kg/day). In this study, it was observed that fucoidans demonstrated the potential to improve the health conditions of microcystin-LR-injected Swiss albino mice by downregulating the levels of pro-inflammatory cytokine levels (IL-1β, IL-6, and TNF-α) in serum [56]. The anti-inflammatory effects of LMW fucoidans (*S. hemiphyllumin*) were also reported in human intestinal epithelial cells (Caco-2 cells) [57]. According to the authors, fucoidans enhanced the immune function of LPS-exposed Caco-2 cells by lowering IL-1β and TNF-α secretion and stimulating anti-inflammatory cytokines. In addition, the pro-inflammatory cytokine inhibitory effects of LMW fucoidans have been reported by Xu et al. (2018). According to the authors, LMW fucoidans, isolated from *S. japonica,* ameliorated the inflammatory response by downregulating *IL-6* and by upregulating *IL-10* transcriptional levels in atherosclerotic mice [58]. In another study, Wang et al. (2016) showed that fucoidans isolated from *L. japonica* had the potential to inhibit pro-inflammatory cytokine secretion (IL-1β, IL-6, and TNF-α) in atherosclerosis in LDLR^-^/^-^ mice [59]. Kim et al. (2018) studied the cytokine profiles of LMW fucoidans in UV-B-exposed six-week female HR-1 hairless mice and found that fucoidan treatment stimulated IL-10 secretion and subsequently reduced the levels of IL-1β in the UV-B exposed groups [60]. Hu et al. (2019) also reported the IL-6 and TNF-α inhibitory properties of fucoidans in 6–8-week-old male SPF SD rats (myocardial infarction model). In addition, LMW fucoidans isolated from *L. japonica* ameliorated peripheral arterial disease in diabetic rats by downregulating IL-1β protein expression [37]. The authors suggested that the oral administration of fucoidans (20–40 mg/kg) for 4 weeks had the potential to reduce the risk of peripheral arterial disease in diabetic rats. The protective effects of fucoidans against radiation-induced cytokine expression (IL-1) in lung tissues were recently reported by Yu et al. (2018), in an 8-week-old male C57BL/6 mouse model [60]. Furthermore, Xu et al. (2019) reported the IL-6 inhibitory effect of LMW fucoidans isolated from *S. japonica,* in a male ApoE-knockout (-/-) mouse model. According to the authors, fucoidans could ameliorate symptoms of atherosclerosis by downregulating the gene and protein expression levels of IL-6 [61].

In addition, Fernando et al. (2017) compared the pro-inflammatory cytokine inhibitory properties of fucoidans isolated from *C. minima* in LPS-activated macrophages. In this study, the authors confirmed the ability of fucoidans to inhibit LPS-activated TNF-α, IL1-β, and IL-6 using ELISA assays [27]. The fucoidans used in this study contained 34% sulfate of its dry weight and approximately 80% fucose of total sugars. In addition, a novel fucose-containing sulfated polysaccharide (→4)-α-D-Galp-(1→ and →3)-β-L-Fucp-(1→) isolated from *S. thunbergii* was found to inhibit LPS-induced *TNF-α* and *IL-6* expression in RAW 264.7 macrophages [28]. Jeong et al. (2016) also reported pro-inflammatory cytokine inhibitory properties of fucoidans, purchased from Sigma-Aldrich, in LPS-activated RAW 264.7 cells. The authors observed that fucoidans inhibited the elevated *TNF-α*, *IL1-β*, and *IL-6* gene expression in LPS-exposed (500 ng/mL) RAW 264.7 cells, without being toxic to the macrophages [22]. Barbosa et al. (2019) also studied the anti-inflammatory effects of fucoidan/chitosan nanoparticles in activated THP-1 monocytes. According to the authors, fucoidan treatment downregulated pro-inflammatory cytokine secretion (IL-1β, IL-6, and TNF-α) in activated THP1 monocytes [62]. Lee et al. (2016) also evaluated the effect of fucoidans in treating chronic kidney disease, using mesenchymal stem cells (MSCs). According to the results, co-culture of LPS-stimulated macrophages with fucoidan-treated MSCs significantly suppressed the expression of TNF-α and upregulated the expression of IL-10. The results observed by Lee et al. (2016) are important, as they reported the pro-inflammatory cytokines inhibitory effects of fucoidans in addition to the anti-inflammatory cytokine stimulatory properties of fucoidans [63]. Kim et al. (2018) described the importance of *TNF-α* inhibition in the suppression of excessive phagocytic capacity of porcine peripheral blood polymorphonuclear cells using LPS-stimulated peripheral polymorphonuclear cells (PMNs). The authors observed the increased phagocytic ability of PMNs culture supernatant after LPS exposure. However, fucoidan (*F. vesiculosus*)-treated cells showed weaker phagocytic capacity, compared with the LPS-treated group [64]. In addition, Liying et al. (2020) reported pro-inflammatory cytokine inhibitory properties of fucoidan isolated from *Saccharina japonica* against LPS-activated RAW 264.7 macrophages. According to the authors, fucoidans (1 → 3 linked α-L-fucopyranosyl and →4-αL fucopyranosyl, with sulfate groups at C–4 and partially at C–2 positions) were capable to inhibit LPS-activated TNF-α, IL-1β, and IL-6 observed in RAW 264.7 macrophages (3.25–25 μg/mL) [65]. Pro-inflammatory cytokine inhibitory effects reported from fucoidans are summarized in Table 3.

### 2.5. Chemokine Inhibition of Fucoidan 

Chemotactic cytokines are usually referred to as chemokines (CHEMOtactic CytoKINES). Chemokines are a family of low molecular weight proteins that coordinate leukocyte trafficking by binding to seven-transmembrane domain receptors. Based on the cysteine residues, chemokines are assigned to four groups: C chemokines (lymphotactin), C-C chemokines (MCP-1, monocyte inflammatory protein [MIP-1α], and MIP-1β), C-X-C chemokines, and CXXXC chemokines [54]. Several studies have shown that fucoidans isolated from brown seaweeds are capable of inhibiting chemokine secretion in inflammatory cells. Here, we discuss the studies that reported the inhibitory effects of fucoidans, isolated from different brown seaweeds, on chemokines.

Chen et al. (2017) reported the CCL2 inhibitory effect of a commercial grade of LMW fucoidans, purchased from a commercial dealer [69]. During the study, the authors noted that fucoidan treatment inhibited CCL2/MCP-1 secretion in HCT 116 cancer cells treated with LMW fucoidans. Although this study did not directly report the anti-inflammatory effect of fucoidans, the results provide useful insights regarding the importance of inhibition of upregulated inflammatory responses to cancer prevention treatment. Dutot et al. (2019) also showed the chemokine inhibitory properties of fucoidans in poly (I:C)-exposed bronchial epithelial cells. According to the authors, fucoidans inhibited the secretion of several chemokines, including CCL5, CCL22, CXCL1, CXCL5, and CXCL8 [66]. However, the results reported by Dutot et al. (2019) were contradictory to those reported by Chen et al. (2017), who stated that the levels of CCL2 and CCL20 in the tested cells were not altered even after treatment with fucoidans. This might be due to the use of different cell types and the different modes of action of fucoidans isolated from different seaweeds. Chemokine inhibitory activities of fucoidans isolated from *Laminaria hyperborea* were reported by Kopplin et al. (2018). The authors demonstrated the inhibitory effects of the isolated fucoidans on several chemokines, such as CXCL10 and CCL5, in a Lepirudin-based human whole blood model. The molecular weight of the isolated fucoidans was 469 kDa and the structure of fucoidans was composed of glycosidic linkages (1→3)-α-L-fuco-pyranose, 1→2- α-L-fuco-pyranose, and 1→4-linked α-L-fuco-pyranose [70]. 

In a study by Sun et al. (2016), the levels of serum CCL22, observed in polarized M2 macrophages, were downregulated after fucoidan treatment. In addition, fucoidans inhibited the phosphorylation and translocation of NF-κB-p65 to the nucleus [52]. Similarly, Wang et al. (2016) demonstrated the *CCL3* (also known as MIP1-α) inhibitory effect of fucoidans in male C57BL/6J mice model, fed a high-fat, high-sucrose diet [71]. The authors claimed that the downregulation of CCL3 resulted in the reduction in the inflammatory response in high glucose stressed-activated pancreatic islet cells and protected the cells against apoptosis. Of note, *Acaudina polypodioides* used in this study is a species of sea cucumber and not seaweed.

### 2.6. JAK-STAT Inhibition of Fucoidan

Janus kinase-signal transducers and activators of transcription (JAK-STATs) are important signal transduction mechanisms used to evaluate the anti-inflammatory mechanisms of natural bioactive compounds [50]. It has been reported that LPS is capable of binding with JAK receptors and subsequently triggering receptor-associated JAK phosphorylation, followed by STAT phosphorylation. The phosphorylation of STATS has the potential to induce transcription of inflammation-related genes, such as proinflammatory cytokines, iNOS, and COX2 [72]. To date, STAT1 and STAT3 have been implicated as key modulators in inflammatory signaling cascades triggered by LPS, interferon-gamma (INF-γ), and other cytokines [73]. Recently, several studies reported the potential of fucoidans to inhibit JAK-STAT signal transduction in LPS-activated inflammatory models. In this section, we discuss several of these studies that reported the JAK-STAT inhibitory effects of fucoidans isolated from different seaweeds (Table 4).

Li et al. (2016) evaluated the protective effect of fucoidans isolated from *F. vesiculosus* against concanavalin A (ConA)-induced acute liver injury in BALB/C mice [67]. The authors noted that fucoidans could inhibit ConA-stimulated JAK2 and STAT1 in BALB/C mice [67]. Similarly, Li et al. (2017) examined the protective effects of fucoidans against hepatic ischemia–reperfusion injury in BALB/C mice and observed that the mice had elevated levels of *p*-JAK2 and *p*-STAT1 protein expression and pro-inflammatory cytokine secretion. However, phosphorylation of JAK2 and STAT1 observed in ischemia–reperfusion injury was downregulated by fucoidans treatment [74]. Rui et al. (2017) also reported the inhibitory effect of fucoidans on the phosphorylation of JAK and STAT3 proteins in prostate cancer cells. Although the authors highlighted the anticancer effect of fucoidans, these results demonstrate the possibility of fucoidans inhibiting JAK-STAT-related inflammation in other organs and healthy cells as well [75].

### 2.7. TLRs’ Inhibition of Fucoidan

Toll-like receptors (TLRs) are a group of pattern recognition receptors found in the outer cell membranes and endosome membranes. TLRs are capable of recognizing metabolites secreted by microorganisms, such as bacteria, viruses, and pathogens. Activation of TLRs in response to microbial metabolites triggers intracellular signaling cascades, including NF-κB. As described earlier, NF-κB is an inducible transcription factor that relates to the production of pro-inflammatory mediators, such as pro-inflammatory cytokines, chemokines, COX2, and iNOS [76].

In a recent study, Dutot et al. (2019) assessed the anti-inflammatory mechanisms of fucoidans against poly (I:C) inflammatory responses in the respiratory tract. The authors observed that treatment with fucoidans led to the downregulation of poly (I:C)-stimulated inflammatory responses in human primary bronchial epithelial cells, which was due to the downregulation of *TLR3* gene expression [66]. Fucoidans isolated from *Padina commersonii* were also found to inhibit LPS-induced TLR activation in RAW 264.7 macrophages [43]. According to the authors, fucoidans treatment downregulated LPS-activated gene expression levels of *TLR2* and *TLR4* in RAW 264.7 macrophages. 

### 2.8. Keap1/NRF2 Stimulation Properties Reported from Fucoidans

Activation of Nrf2/Keap-1 is another mechanism involved in the scavenging of ROS species and NOS, which are generated in the cellular environment during inflammatory events [77]. Several studies have shown that fucoidans have the ability to upregulate Nrf2/Keap-1 activity under stress conditions. Here, we discuss several studies that have highlighted the Nrf2/Keap-1 stimulating properties of fucoidans. 

Ryu and Chung (2016) assessed the Keap1/NRF2 stimulatory properties of fucoidans in a human keratinocyte cell line (HaCaT) and found that incubation of the cells with fucoidans (30 µg/mL) for 12 h led to the upregulation of NRF2 expression levels in the nucleus and downregulation of Keap1 levels. Furthermore, the authors also described the potential of fucoidans to induce antioxidant proteins, such as HO-1 and SOD, using Western blot analysis [78]. Recently, Li et al. (2019) reported that fucoidan treatment can enhance NRF2 translocation from the cytosol to the nucleus as well as *NRF2* expression in hydrogen peroxide-induced oxidative damage in porcine intestinal epithelial cells. In addition, Wang et al. (2018) reported the NRF2 stimulatory properties of fucoidans in an ICR mouse model and HL-7702 cells. The authors found that fucoidans (25–100 µg/mL) had the potential to stimulate *NRF2* expression and translocation in acetaminophen-exposed HL-7702 cells. The authors confirmed the results using immunofluorescence and Western blot analysis [77]. The results reported by Li et al. (2019) and Wang et al. (2018) are useful for future studies, as there is limited literature available regarding the effect of fucoidans on NRF2 signal transduction in the anti-inflammatory process [79].

## 3. Material and Methods

Google Scholar and Scopus databases were searched to screen relevant articles in this review article. Articles reviewed in the background and introduction sections were based on novelty, time of publication, quality of the journals, and the number of citations. Papers published during 2016–2020 were scanned with the keywords “inflammation” and “fucoidan” to locate the anti-inflammatory properties of fucoidan.

## 4. Conclusions

In this study, we reviewed various studies that reported the anti-inflammatory properties of fucoidans isolated from different seaweeds. According to the literature published during 2016–2020, it is clear that fucoidans have the potential to be developed as functional food and pharmaceuticals for the treatment of inflammatory diseases. However, the anti-inflammatory properties of fucoidans seem to depend on the type of seaweed, monosugar composition, and sulfate content. Therefore, the development of pharmaceuticals from fucoidans seems to be a challenging task when compared with other natural metabolites present in seaweeds, such as phlorotannins, sterols, and pigments. Nonetheless, there is tremendous potential to develop fucoidans as a functional food to reduce the risk of chronic inflammatory diseases. 

Future prospective should focus on the production of fucoidan with exact molecular weight, composition, sulfate ester pattern, and content to use fucoidan in commercial applications such as cosmeceuticals and nutraceuticals. Furthermore, fucoidan synthesis by using genetically modified cell lines and further developing steps should optimize production and extraction processes from cultivated brown seaweeds. These steps may ensure the year-round production of fucoidan for industrial applications. Otherwise, anti-inflammatory properties and potential applications discussed with regard to fucoidans will not be achieved in the future.

## Figures and Tables

**Table 1:** COX2 inhibitory properties of fucoidans isolated from brown seaweeds.

Seaweed	Tested Organism	Effect	Reference
*Chnoospora minima*	LPS-activated RAW 264.7 macrophages	COX2 inhibition	[27]
*Chnoospora minima* and *Sargassum polycystum*	LPS-activated RAW 264.7 macrophages	Inhibit LPS-induced PGE2 secretion	[26]
*Commercial grade fucoidan*	Male Wistar albino rats	COX2 inhibition	[24]
*Commercial grade fucoidans*	BALB/c mice and RAW 264.7 macrophages	Inhibit LPS-induced COX2 secretion	[23]
*Fucus vesiculosus, Undaria pinnatifida,* and *Macrocystis pyrifera*	Chondrocytes and Synoviocytes	Inhibit IL-1β-induced COX2 secretion	[21]
*Fucus vesiculosis*	RAW 264.7 macrophages and zebrafish embryos	Inhibit LPS-induced PGE2 secretion	[22]
*Hikizia fusiforme*	RAW 264.7 macrophages	COX2 inhibition	[29]
*Sargassum thunbergii*	RAW 264.7 macrophages	Downregulation of LPS-activated COX2 expression	[28]
*Turbinaria ornate*	RAW 264.7 macrophages and zebrafish embryos	COX2 inhibition	[25]
*Undaria pinnatifida*	Rabbit articular chondrocytes	Downregulation of COX2 observed in chondrocytes	[20]
*Undaria pinnatifida*	Arthritic rats	Downregulation of COX2	[20]

**Table 2:** NF-κB and MAPK inhibitory properties of fucoidans isolated from brown seaweeds.

Seaweed	Tested Organism	Effect	Reference
Commercial grade fucoidan	NOD diabetic mice	NF-κB p65 inhibition	[41]
Commercial fucoidan	Ischemia–reperfusion injured rats	Inhibition of phosphorylation of ERK, JNK, and p38	[47]
Commercial fucoidan	M2 macrophages	NF-κB p65 inhibition	[52]
Commercial fucoidan	Sprague Dawley (SD) rats	Inhibition of phosphorylation of ERK, JNK, and p38	[47]
*Fucus vesiculosus*	Balb/c mice model	Inhibition of phosphorylation of ERK	[51]
*Hizikia fusiforme*	murine microglial BV2 cells	NF-κB p65 inhibition	[42]
*Kjellmaniella crassifolia*	Wistar rat	Inhibition of aspirin-induced NF-κB activation via stabilization of IκB-α	[36]
*Laminaria Japonica*	SPF SD rats	Downregulated IκB degradation	[37]
*Laminaria japonica*	diabetic db/db mice	Downregulated NF-κB degradation	[38]
*Laminaria japonica*	Hyperuricemic mice	NF-κB p65 inhibition	[40]
*Laminaria japonica*	MDA-MB-231 and HCC1806 cells	Inhibition of phosphorylation of ERK, JNK, and p38	[53]
*Padina commersonii*	RAW 264.7 cells	Inhibition of phosphorylation of IKK and subsequent phosphorylation of NF-κB-p65 and p50	[43]
*Sargassum cristaefolium*	RAW 264.7 cells	Inhibition of phosphorylation of ERK, JNK, and p38	[50]
*Sargassum horneri*	RAW 264.7 cells	LPS-activated IKβ-α phosphorylation	[44]
*Sargassum horneri*	RAW 264.7 cells	Inhibition of phosphorylation of ERK and JNK	[43]
*Sargassum weizhouense*	Kunming inbred mice	NF-κB p65 inhibition	[39]
*Undaria pinnatifida*	DU-145 cancer cells	Inhibition of phosphorylation of ERK and p38	[48]

**Table 3:** Cytokine inhibitory properties of fucoidans isolated from brown seaweeds.

Seaweed	Tested Organism	Effect	Reference
*Ascophyllum nodosum*	Bronchial epithelial cells	Inhibition of IL-1β, IL-6, and TNF-α	[66]
*Chnoospora minima*	RAW 264.7 cells	Inhibition of IL-1β, IL-6, and TNF-α	[27]
Commercial fucoidan	Mesenchymal stem cell	Inhibit TNF-α	[63]
*Ecklonia cava*	HR-1 hairless mice	Inhibition of UV-B-exposed IL-1β production	[60]
*Fucus vesiculosus*	RAW 264.7 cells	Inhibition of IL-1β, IL-6, and TNF-α	[22]
*Fucus vesiculosus*	THP1 monocytes	Inhibition of IL-1β, IL-6, and TNF-α	[62]
*Fucus vesiculosus*	Peripheral polymorphonuclear cells	Inhibition of TNF-α	[64]
*Fucus vesiculosus*	BALB/C mice	Inhibition of TNF-α	[67]
*Laminaria Japonica*	Swiss albino mice	Inhibition of IL-1β, IL-6, and TNF-α	[56]
*Laminaria Japonica*	LDLR-/- mice	Inhibition of IL-1β, IL-6, and TNF-α	[59]
*Laminaria Japonica*	SPF SD rats	Inhibition of IL-6 and TNF-α	[37]
*Saccharina japonica*	Atherosclerotic mice	Inhibition of IL-6	[58]
*Saccharina japonica*	ApoE-knockout (-/-) mice	Inhibition of IL-1 expression	[61]
*Sargassum hemiphyllum*	C57BL/6 mice model	Inhibition of IL-1 expression	[60]
*Sargassum hemiphyllum*	Caco-2 cell	Inhibition of IL-1β and TNF-α	[57]
*Sargassum thunbergii*	RAW 264.7 cells	Inhibition of IL-1β, IL-6, and TNF-α	[28]
*Undaria pinnatifida*	Balb/c mice	Inhibition of IL-4	[68]

**Table 4:** Chemokine and JAK-STAT inhibitory properties of fucoidans isolated from brown seaweeds.

Seaweed	Tested Organism	Effect	Reference
* Acaudina molpadioides	C57BL/6J mice	CCL3 inhibiton	[71]
*Ascophyllum nodosum*	Bronchial epithelial cells	CCL5, CCL22, CXCL1, CXCL5, and CXCL8 inhibiton	[66]
Commercial fucoidan	Athymic nude mice	*p*-JAK and *p*-STAT3 inhibition	[75]
Commercial fucoidan	HCT 116 cells	CCL2/MCP-1 inhibiton	[69]
Commercial fucoidan	M2 macrophages	CCL2, CCL4, CCL5 and CCL22 inhibiton	[52]
*Fucus vesiculosis*	Male BALB/C mice	*p*-JAK2 and *p*-STAT1 inhibition	[74]
*Fucus vesiculosis*	BALB/C mice	*p*-JAK2 and *p*-STAT1 inhibition	[67]
*Laminaria hyperborea*	Human whole blood	CXCL10 and CCL5 inhibiton	[70]

* Source of fucoidans is not seaweed.

## Data Availability

The data presented in this study are available on request from the corresponding author.
